# Genetic causal association between physical activities and epilepsy: A Mendelian randomization study

**DOI:** 10.1002/brb3.3463

**Published:** 2024-03-07

**Authors:** Peihong Li, Jiaxin Li, Zheng Xiao, Dandan Sheng, Weiping Liu, Bo Xiao, Luo Zhou

**Affiliations:** ^1^ Department of Neurology, Xiangya Hospital Central South University Changsha Hunan China; ^2^ National Clinical Research Center for Geriatric Disorders, Xiangya Hospital Central South University Changsha Hunan China; ^3^ Department of Pathology First Hospital of Changsha Changsha Hunan China

**Keywords:** epilepsy, Mendelian randomization, physical activities

## Abstract

**Background:**

Despite numerous investigations into the relationship between physical activities (PA) and epilepsy, the causal effects remain contentious. Thus, we conducted a two‐sample Mendelian randomization (MR) study to assess the potential causality.

**Methods:**

Single‐nucleotide polymorphisms (SNPs) predisposed to self‐reported moderate and vigorous physical activities (MPA and VPA) and overall acceleration average (OAA) calculated through wrist‐worn accelerometers were selected as exposure instrumental variables. Five subtypes of epilepsy, including all epilepsy, focal epilepsy and generalized epilepsy (with or without each other), focal epilepsy‐strict definition and generalized epilepsy‐strict definition (without overlap), were chosen as the outcomes. The MR study utilized the inverse‐variance weighted (IVW) method as the primary analytical tool, supplemented by MR‐Egger, simple mode, weighted mode, and weighted median methods. Cochran's Q and MR‐Egger intercept tests were employed to assess heterogeneity and pleiotropy, while MR pleiotropy residual sum and outlier and leave‐one‐out analyses were conducted to identify potential SNP outliers.

**Results:**

The study indicated that OAA was genetically linked to a decreased risk of both focal epilepsy (OR = 0.812, 95% CI: 0.687–0.960, *p* = .015, IVW) and focal epilepsy‐strict definition (OR = 0.732, 95% CI: 0.596–0.900, *p* = .003, IVW; OR = 0.749, 95% CI: 0.573−0.979, *p* = .035, Weighted median). Genetically predicted MPA and VPA did not exhibit a causal association with all epilepsy or its subtypes (*p*>.05). No evidence of heterogeneity, pleiotropy, or SNP outlier was observed.

**Conclusions:**

Our findings suggested that PA with accelerometer monitoring may potentially reduce the risk of focal epilepsy, while there is no evidence supporting causal association between self‐reported MPA or VPA and either focal or generalized epilepsy.

## Graphical abstract

We conducted a two‐sample Mendelian randomization study to assess the potential causality between physical activities and epilepsy. Self‐reported moderate and vigorous physical activities and overall acceleration average calculated through wrist‐worn accelerometers were selected as the exposure traits. Five subtypes of epilepsy, including all epilepsy, focal epilepsy and generalized epilepsy (with or without each other), focal epilepsy‐strict definition and generalized epilepsy‐strict definition (without overlap), were chosen as the outcome traits. Our findings suggested that physical activities with accelerometer monitoring may potentially reduce the risk of focal epilepsy.

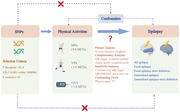

## 1 INTRODUCTION

Epilepsy stands as a prevalent neurological disorder marked by paroxysmal, unprovoked, and recurrent epileptic seizures, impacting nearly 50 million individuals globally (Anon, 2019). Given its chronic nature, epilepsy and its associated comorbidities impose substantial financial strains on both society and families. Thus, the quest for and identification of modifiable risk factors to preclude the onset of epilepsy holds paramount significance (Nakken et al., 2005).

The role of physical activities (PA) has garnered considerable attention for its potential in enhancing overall health (Haskell et al., 2007) and mitigating the adverse effects of certain neurological disorders such as stroke (Kramer et al., 2019), dementia (Lamb et al., 2018), and Parkinson's disease (Tsukita et al., 2022). While numerous studies have endeavored to explore the relationship between PA and epilepsy, the question of whether PA might causally influence the risk of epileptic seizures remains a subject of debate (Carrizosa‐Moog et al., 2018; Dunabeitia et al., 2022; van den Bogard et al., 2020). A multicenter study involving 1677 patients, utilizing a closed‐ended questionnaire to ascertain the most commonly reported triggers of seizures, identified PA as a potential precipitant of epileptic seizures (Nakken et al., 2005). Additionally, another investigation revealed a notably higher incidence of epileptic seizures provoked by PA in individuals afflicted with Dravet syndrome (Verbeek et al., 2015). Nonetheless, conflicting findings exist within the literature. Livingston et al. (1978) reported no instances of seizure relapse or exacerbation associated with exercise across a cohort of 15,000 epilepsy patients monitored over a 36‐year follow‐up period. Similarly, a prior randomized controlled trial employing a parallel‐group design and blinded outcome assessment, encompassing 117 individuals with epilepsy, of whom 58 were allocated to an exercise intervention group and the remainder to a control group, demonstrated a marginal reduction in mean seizure frequency during follow‐up in both cohorts, albeit statistically nonsignificant (Kumar et al., 2022). Consequently, the causal relationship between PA and the risk of epilepsy characterized by recurrent seizures remains ambiguous.

Mendelian randomization (MR) serves as a widely employed method for discerning the causal connection between risk factors and disease outcomes (Sekula et al., 2016). By utilizing genetic instrumental variables (IVs), MR allows for the assessment of associations between exposures and outcomes while mitigating the influence of confounding factors, thus potentially enhancing the robustness of derived causal relationships (Davey Smith & Hemani, 2014). In this study, we conducted a two‐sample MR analysis to investigate the causal association between PA and epilepsy.

## 2 MATERIALS AND METHODS

### 2.1 Study design and data source

We conducted a MR analysis to investigate the causal relationship between the modifiable exposure factors of PA and epilepsy. To ascertain the validity of genetic variants as IVs, three fundamental conditions must be satisfied in MR analysis: first, the chosen IVs should exhibit a significant association with PA; second, they should not be linked to any confounding factors of PA and epilepsy. Moreover, the selected IVs should exclusively affect epilepsy through their influence on PA (Boef et al., 2015).

The exposure data utilized in this study were derived from a recent genome‐wide association study (GWAS) conducted by the MRC Integrative Epidemiology Unit at the University of Bristol, encompassing more than 440,200 European participants. In this investigation, number of days per week engaging moderate physical activities (MPA) and vigorous physical activities (VPA) more than 10 minutes served as self‐reported variables collected via a touchscreen‐based questionnaire, representing the exposure traits. Metabolic equivalent (MET) is a standardized unit quantifying the rate of oxygen consumption by the body at rest, defined as 3.5 mL/(kg·min) of oxygen consumption. It serves to quantify the energy expenditure associated with PA by expressing it in multiples of MET. MPA was delineated as PA ranging between 3 and 6 METs, while VPA was characterized by activities exceeding 6 METs (Ainsworth et al., 2011). Diverging from self‐reported data, the overall acceleration average (OAA), PA measurement computed through Axivity AX3 wrist‐worn triaxial accelerometers in another GWAS comprising 377,234 individuals of alike European descent, was selected as an alternative exposure trait. In this study, OAA is defined by accelerations below 425 milli‐gravities, corresponding to an intensity equivalent to 6 METs (Klimentidis et al., 2018).

**FIGURE 1:**
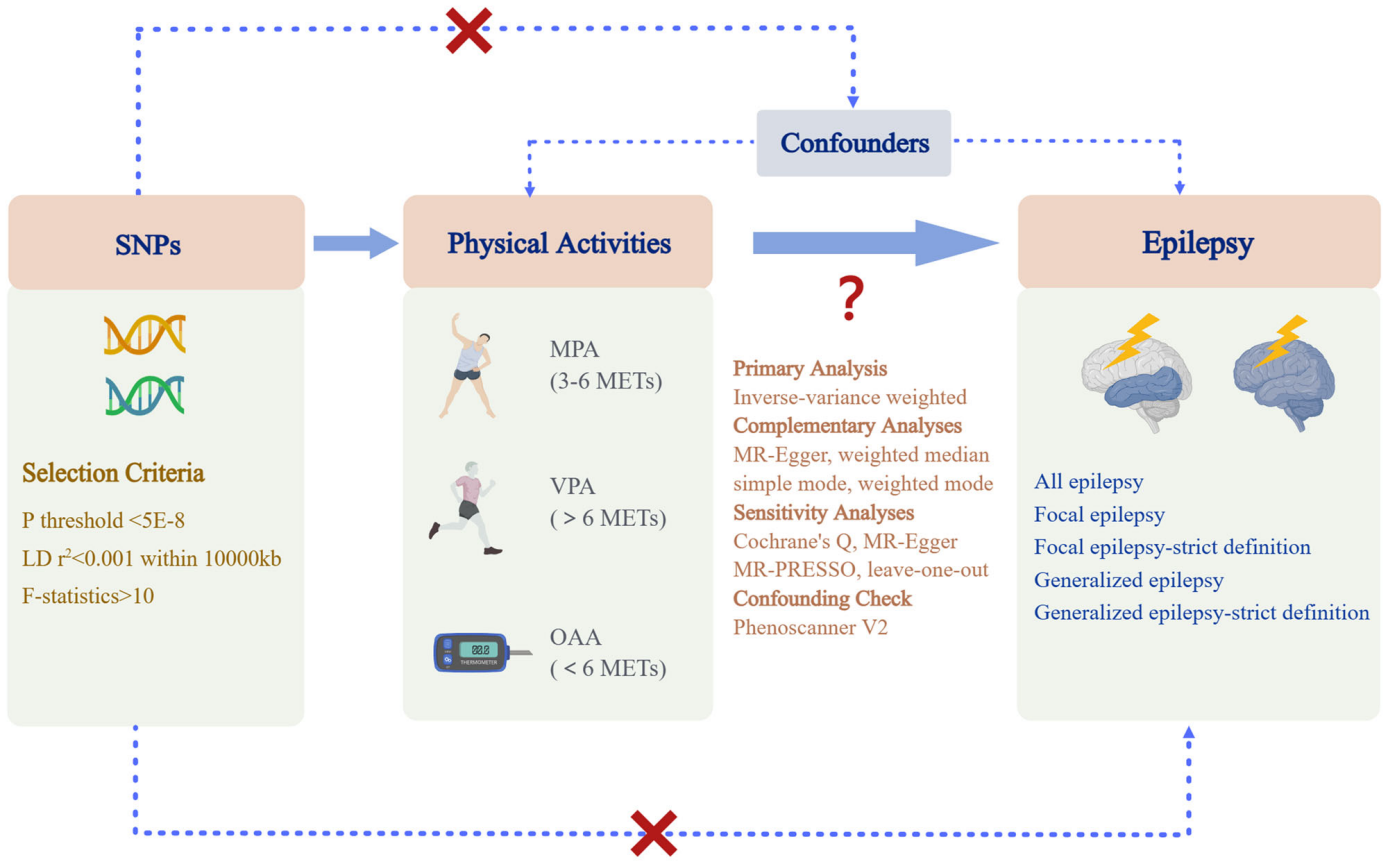
**Study design overview**. SNPs, single‐nucleotide polymorphisms; LD, linkage disequilibrium; MPA, moderate physical activities; VPA, vigorous physical activities; OAA, overall acceleration average; METs, metabolic equivalents; MR‐PRESSO, Mendelian randomization pleiotropy residual sum and outlier; MR, Mendelian randomization.

The outcome traits for this study were sourced from the FinnGen study, conducted in Finland, which comprised participants of European descent and encompassed genotypic and digital healthcare data from over 589,000 individuals. Five distinct phenotypes of disease outcomes were selected, including all epilepsy, focal epilepsy and generalized epilepsy (with or without each other), as well as focal epilepsy‐strict definition and generalized epilepsy‐strict definition (without overlap). Detailed information regarding the summary data for exposure and outcome traits is presented in Table 1. The utilization of publicly available data obviated the necessity for ethics approval and consent to participate. The study's workflow is depicted in Figure 1.

**TABLE 1:** Summary of data source of of physical activities and epilepsy.

Datasets	Ancestry	Sample size	Cases	Controls	nSNPs	Author or consortium	GWAS ID
**Exposures**							
MPA	European	440,266	/	/	9,851,867	MRC‐IEU	ukb‐b‐4710
VPA	European	440,512	/	/	9,851,867	MRC‐IEU	ukb‐b‐151
OAA	European	91,084	/	/	11,796,201	Klimentidis YC	ebi‐a‐GCST006099
**Outcomes**							
EP	European	182,367	6260	176,107	16,380,349	FinnGen	finn‐b‐G6_EPLEPSY
FE	European	213,461	929	212,532	16,380,452	FinnGen	finn‐b‐FE
GE	European	214,313	1781	212,532	16,380,451	FinnGen	finn‐b‐GE
FE‐ST	European	213,143	611	212,532	16,380,452	FinnGen	finn‐b‐FE_STRICT
GE‐ST	European	213,995	1463	212,532	16,380,451	FinnGen	finn‐b‐GE_STRICT

nSNPs, number of single‐nucleotide polymorphisms; GWAS, genome‐wide association study; MPA, moderate physical activities; VPA, vigorous physical activities; OAA, overall acceleration average; EP, epilepsy; FE, focal epilepsy; GE, generalized epilepsy; FE‐ST, focal epilepsy‐strict definition; GE‐ST, generalized epilepsy‐strict definition.

### 2.2 Instrumental variables selection

Single‐nucleotide polymorphisms (SNPs) genetically predisposed to MPA, VPA, and OAA were chosen as IVs. The selection criteria for IVs included the absence of linkage disequilibrium (pairwise *r* ^2 ^< 0.001 within 10,000 kb) and surpassing the genome‐wide significance threshold of *p* < 5E‐08. SNPs with a minor allele frequency less than 0.01 were excluded due to potential low confidence. To address the bias of weak genetic instruments, all IVs associated with exposure traits analyzed in this study had a minimum *F*‐statistic of 10 (Haycock et al., 2016). PhenoScanner was utilized to check all IVs and the one associated with confounding factors should be removed. Subsequently, the effects of SNPs on exposures and outcomes were aligned, except for palindromic SNPs with intermediate allele frequencies.

In total, 18, 10, and 8 SNPs were identified as IVs for MPA, VPA, and OAA, respectively (further details provided in Table S1).

### 2.3 Mendelian randomization and statistical analyses

The inverse‐variance weighted (IVW) method, widely acknowledged for its robustness in MR studies, was selected as the primary analytical approach to estimate the odds ratio (OR) and *p* value (Burgess et al., 2017). Additionally, MR‐Egger, simple mode, weighted mode, and weighted median methods were employed as supplementary analyses. All statistical computations were conducted using the “TwoSampleMR” package (Version 0.5.7) within RStudio (Version 2023.06.0). Given the exploratory nature of our investigation, a Bonferroni correction was not applied. The significance threshold was set at *p* < 0.05.

### 2.4 Sensitivity analyses

Cochran's Q and MR‐Egger intercept tests were utilized to evaluate the existence of heterogeneity and pleiotropy among the IVs. Additionally, MR pleiotropy residual sum and outlier (MR‐PRESSO) analysis, coupled with leave‐one‐out analysis, were performed to identify potential outliers among the IVs (Greco et al., 2015; Verbanck et al., 2018). In cases where an outlier was identified by the MR‐PRESSO or leave‐one‐out, it should be excluded in MR analysis.

## 3 RESULTS

**FIGURE 2:**
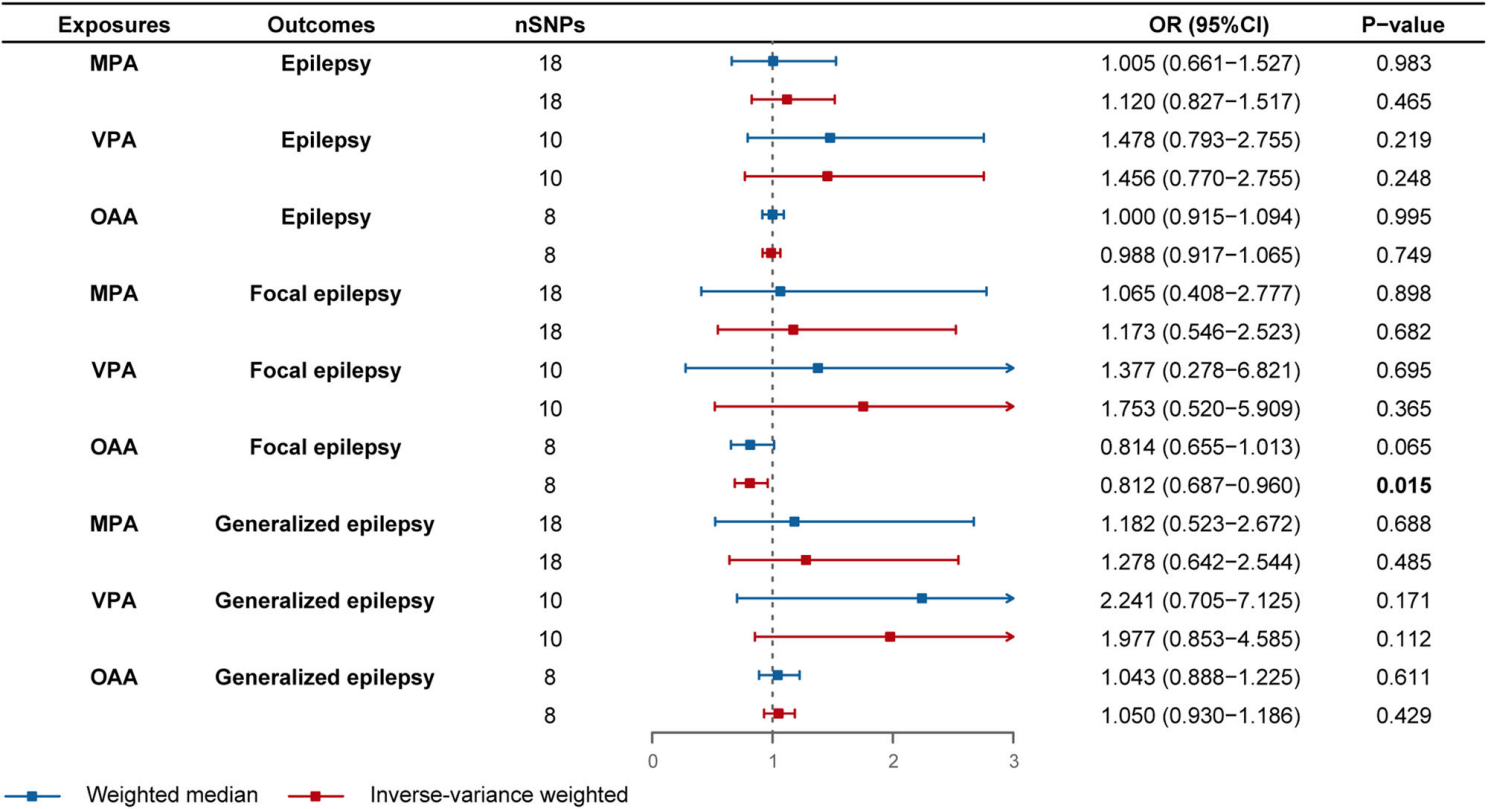
Forest plot for the causal effect of physical activities on the risk of all epilepsy, focal and generalized epilepsy (with or without each other). nSNPs, number of single‐nucleotide polymorphisms; OR, odds ratio; CI, confidence interval; MPA, moderate physical activities; VPA, vigorous physical activities; OAA, overall acceleration average.

**FIGURE 3:**
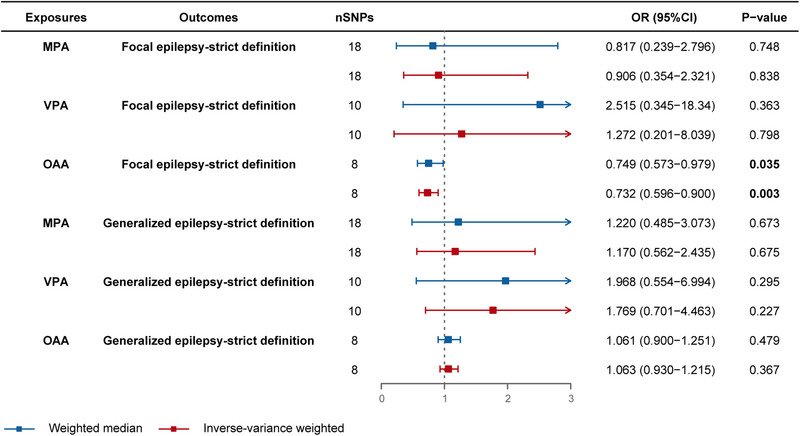
Forest plot for the causal effect of physical activities on the risk of focal epilepsy‐strict definition and generalized epilepsy‐strict definition (without overlap). nSNPs, number of single‐nucleotide polymorphisms; OR, odds ratio; CI, confidence interval; MPA, moderate physical activities; VPA, vigorous physical activities; OAA, overall acceleration average.

**FIGURE 4:**
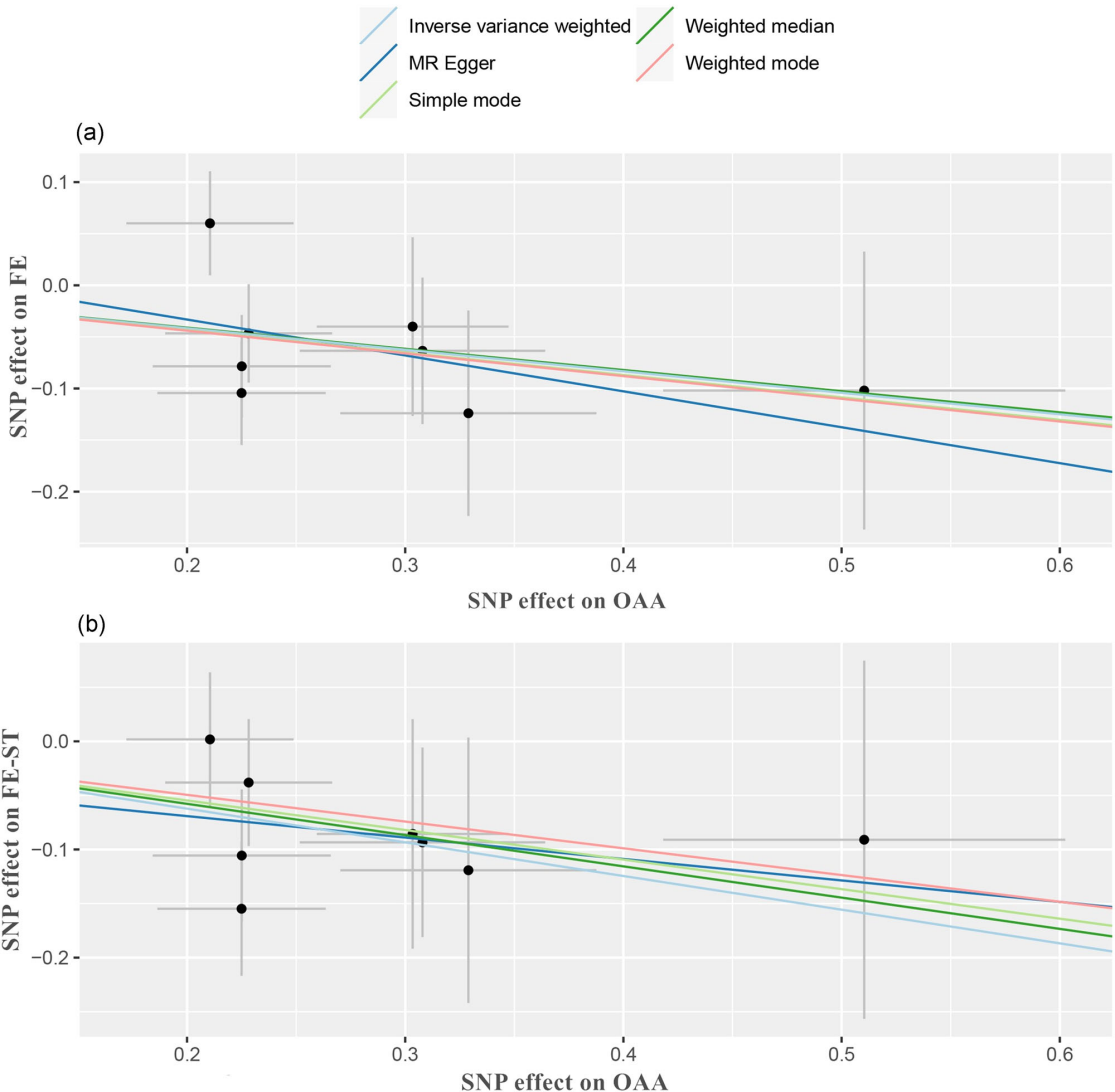
Scatter plots for the significant causal effects of overall acceleration average calculated through wrist‐worn accelerometers on the risk of (A) focal epilepsy and (B) focal epilepsy‐strict definition. SNP, single‐nucleotide polymorphism; OAA, overall acceleration average; FE, focal epilepsy; FE‐ST, focal epilepsy‐strict definition; MR, Mendelian randomization.

Using the two‐sample MR approach with IVW estimation, we successfully established a causal link between OAA and a diminished risk of focal epilepsy (OR = 0.812, 95% CI: 0.687–0.960, *p* = 0.015) (Figure 2). Subsequently, we proceeded to stratify the outcome subtypes into focal epilepsy‐strict definition and generalized epilepsy‐strict definition, wherein OAA demonstrated a causal association with reduced risk of focal epilepsy‐strict definition (OR = 0.732, 95% CI: 0.596–0.900, *p* = 0.003, IVW; OR = 0.749, 95% CI: 0.573−0.979, *p* = 0.035, weighted median) (Figure 3). Thus, specific PA involving acceleration may potentially confer a protective effect against focal epilepsy (Figure 4). Conversely, genetically predicted MPA and VPA did not exhibit any causal association with epilepsy or its subtypes (*p* > 0.05) (Figures 2 and 3). Notably, our findings underscored that engaging in PA did not elevate the risk of developing either focal or generalized epilepsy. The results from all five MR methods were presented in Tables S2 and S3.

**FIGURE 5:**
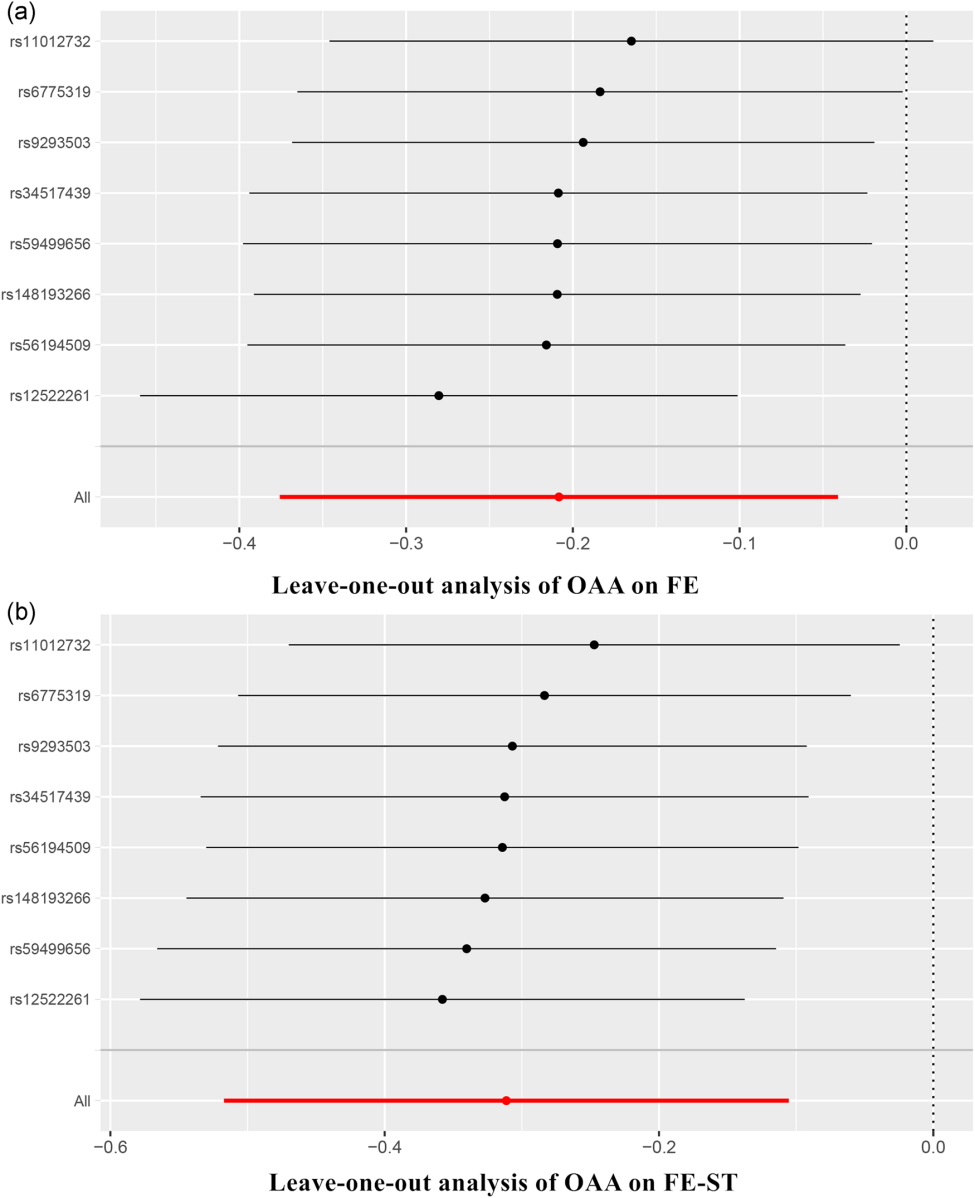
Leave‐one‐out analysis yielded no SNP outlier for causal effects of overall acceleration average calculated through wrist‐worn accelerometers on (A) focal epilepsy and (B) focal epilepsy‐strict definition. SNP, single‐nucleotide polymorphism; OAA, overall acceleration average; FE, focal epilepsy; FE‐ST, focal epilepsy‐strict definition.

For sensitivity analyses, the validity of the IVs and assumptions of the MR model were verified using Cochran's *Q* test and MR‐Egger intercept test. A statistically significant heterogeneity of IVs was observed with a *p* value of 0.044 in Cochran's *Q* test assessing the impact of VPA on epilepsy. To address this issue, we employed the random‐effects model of the IVW method for statistical analysis. Conversely, no evidence of heterogeneity or pleiotropy was observed in the other findings. Neither the MR‐PRESSO nor leave‐one‐out analysis (Figure 5) detected any potential instrumental outliers (Table 2). Therefore, our sensitivity analyses provided robust support for our experimental results.

**TABLE 2:** Sensitivity analyses of the causal association between physical activities and the risk of epilepsy.

		Heterogeneity test (Cochran's *Q*)			Horizontal pleiotropy			MR‐PRESSO
Exposures	Outcomes	*Q*	*Q* df	*p* Value	Egger intercept	SE	*p* Value	*p* Value
MPA	EP	15.609	17	.552	−0.014	0.032	.662	.539
VPA	EP	17.307	9	.044	−0.108	0.067	.147	.055
OAA	EP	8.942	7	.257	−0.007	0.047	.895	.227
MPA	FE	5.122	17	.997	−0.062	0.081	.451	.997
VPA	FE	9.851	9	.363	−0.039	0.146	.795	.381
OAA	FE	6.345	7	.500	0.036	0.100	.728	.519
MPA	GE	26.133	17	.072	0.033	0.075	.667	.086
VPA	GE	6.149	9	.725	0.036	0.096	.716	.746
OAA	GE	4.973	7	.663	0.023	0.071	.753	.697
MPA	FE‐ST	6.744	17	.987	−0.107	0.099	.296	.989
VPA	FE‐ST	15.019	9	.090	−0.126	0.218	.579	.098
OAA	FE‐ST	3.896	7	.792	−0.029	0.121	.815	.810
MPA	GE‐ST	24.507	17	.106	0.035	0.080	.667	.136
VPA	GE‐ST	7.140	9	.623	0.016	0.105	.885	.640
OAA	GE‐ST	5.486	7	.601	−0.007	0.078	.934	.640

MR‐PRESSO, Mendelian randomization pleiotropy residual sum and outlier; SE, standard error; MPA, moderate physical activities; VPA, vigorous physical activities; OAA, overall acceleration average; EP, epilepsy; FE, focal epilepsy; GE, generalized epilepsy; FE‐ST, focal epilepsy‐strict definition; GE‐ST, generalized epilepsy‐strict definition.

## 4 DISCUSSION

In this study, we employed the MR approach to assess potential causal association between PA and epilepsy. Our findings indicated that PA with accelerometer monitoring equivalent to moderate intensity may confer a protective effect, causally lowering the risk of focal epilepsy. This suggested that engaging in sports such as running, race walking, cycling, and ball games could potentially reduce the occurrence of focal epileptic seizures. It is noteworthy that self‐reported data on PA may be subject to bias. However, the utilization of digital wrist‐worn accelerometers for objective measurement offers a more accurate assessment of an individual's level of PA.

Some literature has suggested that PA may potentially increase the occurrence of epileptic seizures due to metabolic acidosis and elevated body temperature (Agnieszka Stanuszek et al., 2015). However, there is a growing body of epidemiological evidence supporting the notion that PA does not elevate the risk of epileptic seizures but may instead confer a protective effect. For instance, an animal experiment reported a reduction in seizure frequency in rats with temporal lobe epilepsy following an aerobic physical program induced by pilocarpine hydrochloride (Arida et al., 1999). Similarly, a clinical trial by Vancini et al. (2010) investigated electroencephalographic responses to exhaustive acute PA in individuals with temporal lobe epilepsy, revealing that physical exertion and subsequent recovery states were associated with decreased epileptiform discharges compared to the resting state. Furthermore, a randomized clinical trial involving epilepsy patients, with 10 patients in the exercise group and 10 in the control group, demonstrated a significant reduction in the frequency of epileptic seizures in the exercise group. Additionally, improvements in quality of life were noted in the exercise group, particularly in domains such as seizure worry, general quality of life, energy, fatigue, and cognitive function. Moreover, the stress level in the exercise group significantly decreased (Hafele et al., 2021). These findings suggested that engaging in tailored and appropriate PA may be advantageous for individuals at lower risk of epileptic seizures.

The potential mechanisms underlying the protective effects of PA in epilepsy remained incompletely understood. One plausible mechanism involved the regulation of inflammation. First, PA has been associated with a significant increase in T‐regulatory cells, disrupting the Th1/Th2 balance by reducing Th1 cell production. Moreover, PA has been shown to stimulate the secretion of interleukin‐6, which in turn reduces the inflammatory response by up‐regulating interleukin‐10 and inhibiting interleukin‐1β secretion (Sharif et al., 2018). Given the intricate relationship between epilepsy and chronic inflammation, PA held promise as a potential therapeutic intervention. Second, PA may modulate hormone levels. Notably, estrogen, a sex steroid hormone, has been implicated in proconvulsant effects, with reductions in estrogen levels associated with decreased epileptic discharge (Tauboll et al., 2015). Research by Zhuang et al. (2020) indicated that PA was associated with a decrease in estradiol levels, potentially providing relief to individuals and reducing the risk of epilepsy. Third, PA may induce changes in neuroplasticity by promoting motor learning and neuronal function, fostering a conducive environment in brain regions involved in motor control (Nicolini et al., 2021). Brain‐derived neurotrophic factor (BDNF), a neurotrophin known for its role in brain regeneration and injury repair, played a protective role in reducing epileptic seizures by modulating neuroplasticity (Bechara & Kelly, 2013). Inhibiting the receptors of insulin‐like growth factor 1 (IGF‐1) could attenuate the upregulation of BDNF, suggesting that IGF‐1 in response to PA may facilitate the elevation of BDNF and thus exert a neuroprotective effect on the brain (Ding et al., 2006). Another mechanism involved oxidative stress (Hafele et al., 2021), characterized by excessive production of reactive oxygen species that overwhelm antioxidant defenses (Pisoschi & Pop, 2015). Oxidative stress may induce neuronal hyperexcitability and trigger epileptic seizures by impairing neurotransmitter release, ion channel function, and mitochondrial activity. An animal model experiment in epileptic mice provided evidence that moderate‐intensity aerobic PA may enhance antioxidant activity, thereby reducing oxidative stress in the hippocampus and lowering the risk of epilepsy (Feter et al., 2019). In summary, overall acceleration average (OAA), a variable related to PA obtained through professional monitoring equipment, may mitigate the risk of focal epilepsy seizures (including focal epilepsy‐strict definition) through the aforementioned pathways.

Several limitations warrant discussion in our study. First, as the SNPs used in this MR analysis were derived from GWAS conducted primarily in individuals of European descent, it is imperative to validate the relevance of our findings in other ethnic groups. Second, larger‐scale studies with expanded sample sizes are necessary to enhance the robustness of our results. Third, while we categorized PA based on different intensities in this study, more specific and refined classifications should be considered. Each subtype of PA possesses unique characteristics that may lead to varying effects, thus warranting a more nuanced approach. Additionally, it is essential to introduce additional categories of epilepsy, taking into account factors such as etiology (e.g., idiopathic, hereditary, structural, and metabolic), to facilitate further discussion and analysis.

## 5 CONCLUSIONS

In conclusion, our study offered novel evidence suggesting that PA with accelerometer monitoring may potentially reduce the risk of focal epilepsy. However, we did not find evidence supporting a causal association between MPA or VPA and the risk of either focal or generalized epilepsy. Encouraging individuals to participate in PA is recommended, as it does not appear to increase the overall risk of epilepsy.

## AUTHOR CONTRIBUTIONS

**Peihong Li**: Methodology; writing—original draft; writing—review and editing. **Jiaxin Li**: Software; writing—review and editing. **Zheng Xiao**: Software; writing—review and editing. **Dandan Sheng**: Methodology; writing—original draft. **Weiping Liu**: Validation; writing—review and editing. **Bo Xiao**: Conceptualization; writing—review and editing. **Luo Zhou**: Conceptualization; project administration; writing—review and editing; writing—original draft.

## FUNDING

This work was supported by the following funding: National Natural Science Foundation of China (Grant No. 81601139) and Natural Science Foundation of Hunan Province (Grant No. 2017JJ3500).

## CONFLICT OF INTEREST STATEMENT

The authors declare no competing interests.

### PEER REVIEW

The peer review history for this article is available at https://publons.com/publon/10.1002/brb3.3463.

## Supporting information


**Supplementary Table S1**. Details of instrumental variables.

**Supplementary Table S2**. The causal effects of physical activities on the risk of all epilepsy, focal epilepsy, and generalized epilepsy (with or without each other).

**Supplementary Table S3**. The causal effects of physical activities on the risk of focal epilepsy‐strict definition and generalized epilepsy‐strict definition (without overlap).

## Data Availability

The datasets supporting this study are available from IEU OpenGWAS (https://gwas.mrcieu.ac.uk) and the FinnGen consortium (https://www.finngen.fi).
