# Supplementary material for: Genetic causal association between physical activities and epilepsy: A Mendelian randomization study
Source: Brain Behav. 2024 Mar 7;14(3):e3463. doi: 10.1002/brb3.3463 (PMC10918602; doi:10.1002/brb3.3463)
Supplement: Supplementary file 1 — Supplementary Table S1. Details of instrumental variables. [file BRB3-14-e3463-s002.docx]

**Supplementary Table S1**. Details of [instrumental variable](javascript:;)s.

| **Exposures** | **SNPs** | **Chr** | **Position** | **EA** | **OA** | **EAF** | **β** | **SE** | ***P*-value** | **F** |
| --- | --- | --- | --- | --- | --- | --- | --- | --- | --- | --- |
| MPA | rs10098073 | 8 | 143309504 | A | C | 0.473 | 0.031 | 0.005 | 9.60E-10 | 37.403 |
| MPA | rs1036800 | 4 | 46433260 | T | A | 0.789 | -0.034 | 0.006 | 3.70E-08 | 30.281 |
| MPA | rs11749912 | 5 | 88065628 | G | A | 0.574 | 0.029 | 0.005 | 9.40E-09 | 32.953 |
| MPA | rs11913445 | 22 | 20142513 | A | C | 0.168 | -0.037 | 0.007 | 3.40E-08 | 30.459 |
| MPA | rs2246122 | 13 | 44826508 | C | T | 0.551 | 0.028 | 0.005 | 1.40E-08 | 32.180 |
| MPA | rs3094622 | 6 | 30327952 | G | A | 0.135 | -0.054 | 0.007 | 1.70E-13 | 54.329 |
| MPA | rs3129962 | 6 | 32379383 | A | G | 0.129 | -0.045 | 0.007 | 1.30E-09 | 36.884 |
| MPA | rs34555420 | 6 | 26090270 | T | G | 0.098 | -0.046 | 0.008 | 2.80E-08 | 30.840 |
| MPA | rs34775997 | 15 | 95304276 | A | G | 0.139 | -0.040 | 0.007 | 3.10E-08 | 30.647 |
| MPA | rs404907 | 3 | 41187707 | G | A | 0.539 | 0.031 | 0.005 | 4.80E-10 | 38.776 |
| MPA | rs4129572 | 7 | 133636888 | C | T | 0.596 | 0.036 | 0.005 | 2.80E-12 | 48.817 |
| MPA | rs429358 | 19 | 45411941 | C | T | 0.154 | 0.043 | 0.007 | 6.10E-10 | 38.286 |
| MPA | rs4540651 | 1 | 154128672 | A | G | 0.512 | 0.028 | 0.005 | 1.50E-08 | 32.020 |
| MPA | rs4886868 | 15 | 74353561 | G | T | 0.585 | 0.030 | 0.005 | 2.60E-09 | 35.446 |
| MPA | rs7229874 | 18 | 6106730 | A | C | 0.218 | -0.034 | 0.006 | 2.20E-08 | 31.342 |
| MPA | rs7565480 | 2 | 200868926 | G | A | 0.661 | -0.030 | 0.005 | 1.80E-08 | 31.678 |
| MPA | rs9533455 | 13 | 43892830 | T | C | 0.312 | -0.031 | 0.005 | 8.50E-09 | 33.166 |
| MPA | rs997467 | 2 | 199182704 | C | T | 0.572 | 0.031 | 0.005 | 9.50E-10 | 37.417 |
|  |  |  |  |  |  |  |  |  |  |  |
| VPA | rs1491872 | 11 | 27792891 | T | C | 0.347 | 0.025 | 0.004 | 6.30E-09 | 33.725 |
| VPA | rs2005617 | 9 | 33791164 | C | T | 0.625 | 0.025 | 0.004 | 4.50E-09 | 34.380 |
| VPA | rs2189464 | 7 | 8633758 | T | C | 0.248 | -0.027 | 0.005 | 2.50E-08 | 31.031 |
| VPA | rs2764261 | 6 | 108927842 | G | A | 0.626 | -0.028 | 0.004 | 6.90E-11 | 42.552 |
| VPA | rs328900 | 7 | 35020280 | T | C | 0.315 | 0.029 | 0.004 | 6.40E-11 | 42.687 |
| VPA | rs382210 | 3 | 84966018 | A | G | 0.655 | 0.028 | 0.004 | 9.40E-11 | 41.939 |
| VPA | rs429358 | 19 | 45411941 | C | T | 0.154 | 0.039 | 0.006 | 1.10E-11 | 46.057 |
| VPA | rs6533635 | 4 | 113612872 | A | G | 0.624 | 0.026 | 0.004 | 2.10E-09 | 35.891 |
| VPA | rs7072776 | 10 | 22032942 | G | A | 0.722 | 0.027 | 0.005 | 3.60E-09 | 34.854 |
| VPA | rs7749823 | 6 | 26158079 | C | A | 0.149 | -0.035 | 0.006 | 2.20E-09 | 35.825 |
|  |  |  |  |  |  |  |  |  |  |  |
| OAA | rs11012732 | 10 | 21830104 | G | A | 0.332 | -0.225 | 0.039 | 5.40E-09 | 34.036 |
| OAA | rs12522261 | 5 | 152054825 | A | G | 0.343 | -0.211 | 0.038 | 3.90E-08 | 30.207 |
| OAA | rs148193266 | 11 | 104528681 | C | A | 0.043 | 0.510 | 0.092 | 3.10E-08 | 30.671 |
| OAA | rs34517439 | 1 | 78450517 | A | C | 0.121 | -0.308 | 0.056 | 4.40E-08 | 29.972 |
| OAA | rs56194509 | 17 | 43844559 | G | T | 0.22 | 0.303 | 0.044 | 5.00E-12 | 47.677 |
| OAA | rs59499656 | 18 | 40768309 | T | A | 0.344 | 0.228 | 0.038 | 2.40E-09 | 35.598 |
| OAA | rs6775319 | 3 | 18758501 | T | A | 0.729 | -0.225 | 0.041 | 3.50E-08 | 30.431 |
| OAA | rs9293503 | 5 | 87948962 | C | T | 0.112 | -0.329 | 0.059 | 2.10E-08 | 31.420 |

SNPs, single-nucleotide polymorphisms; Chr, chromosome; EA, effect allele; OA, other allele; EAF, effect allele frequency; β, the per-allele effect on physical activity measurement; SE standard error; MPA, moderate physical activities; VPA, vigorous physical activities; OAA, overall acceleration average.
