# Supplementary material for: Genetic causal association between physical activities and epilepsy: A Mendelian randomization study
Source: Brain Behav. 2024 Mar 7;14(3):e3463. doi: 10.1002/brb3.3463 (PMC10918602; doi:10.1002/brb3.3463)
Supplement: Supplementary file 2 — Supplementary Table S2. The causal effects of physical activities on the risk of all epilepsy, focal epilepsy, and generalized epilepsy (with or without each other). [file BRB3-14-e3463-s001.docx]

**Supplementary Table S2**. The causal effects of physical activities on the risk of all epilepsy, focal epilepsy, and generalized epilepsy (with or without each other).

| **Exposures** | **Outcomes** | **Methods** | **SNPs** | **OR** | **95%LCI** | **95%UCI** | ***P-*value** |
| --- | --- | --- | --- | --- | --- | --- | --- |
| MPA | EP | MR Egger | 18 | 1.712 | 0.258 | 11.347 | 0.585 |
|  |  | Weighted median | 18 | 1.005 | 0.661 | 1.527 | 0.983 |
|  |  | IVW | 18 | 1.120 | 0.827 | 1.517 | 0.465 |
|  |  | Simple mode | 18 | 0.959 | 0.473 | 1.946 | 0.909 |
|  |  | Weighted mode | 18 | 0.943 | 0.468 | 1.899 | 0.872 |
| VPA | EP | MR Egger | 10 | 63.681 | 0.611 | 6.6E+03 | 0.118 |
|  |  | Weighted median | 10 | 1.478 | 0.793 | 2.755 | 0.219 |
|  |  | IVW | 10 | 1.456 | 0.770 | 2.755 | 0.248 |
|  |  | Simple mode | 10 | 1.974 | 0.690 | 5.653 | 0.237 |
|  |  | Weighted mode | 10 | 1.917 | 0.652 | 5.642 | 0.267 |
| OAA | EP | MR Egger | 8 | 1.013 | 0.703 | 1.459 | 0.948 |
|  |  | Weighted median | 8 | 1.000 | 0.915 | 1.094 | 0.995 |
|  |  | IVW | 8 | 0.988 | 0.917 | 1.065 | 0.749 |
|  |  | Simple mode | 8 | 0.994 | 0.842 | 1.172 | 0.941 |
|  |  | Weighted mode | 8 | 1.001 | 0.832 | 1.203 | 0.996 |
| MPA | FE | MR Egger | 18 | 7.442 | 0.064 | 861.397 | 0.420 |
|  |  | Weighted median | 18 | 1.065 | 0.408 | 2.777 | 0.898 |
|  |  | IVW | 18 | 1.173 | 0.546 | 2.523 | 0.682 |
|  |  | Simple mode | 18 | 0.888 | 0.174 | 4.543 | 0.889 |
|  |  | Weighted mode | 18 | 0.921 | 0.199 | 4.251 | 0.917 |
| VPA | FE | MR Egger | 10 | 6.941 | 2.8E-04 | 1.7E+05 | 0.717 |
|  |  | Weighted median | 10 | 1.377 | 0.278 | 6.821 | 0.695 |
|  |  | IVW | 10 | 1.753 | 0.520 | 5.909 | 0.365 |
|  |  | Simple mode | 10 | 5.853 | 0.327 | 104.639 | 0.260 |
|  |  | Weighted mode | 10 | 3.938 | 0.239 | 64.877 | 0.363 |
| OAA | FE | MR Egger | 8 | 0.706 | 0.327 | 1.523 | 0.409 |
|  |  | Weighted median | 8 | 0.814 | 0.655 | 1.013 | 0.065 |
|  |  | IVW | 8 | 0.812 | 0.687 | 0.960 | **0.015** |
|  |  | Simple mode | 8 | 0.805 | 0.588 | 1.100 | 0.216 |
|  |  | Weighted mode | 8 | 0.803 | 0.586 | 1.099 | 0.212 |
| MPA | GE | MR Egger | 18 | 0.483 | 0.006 | 39.543 | 0.750 |
|  |  | Weighted median | 18 | 1.182 | 0.523 | 2.672 | 0.688 |
|  |  | IVW | 18 | 1.278 | 0.642 | 2.544 | 0.485 |
|  |  | Simple mode | 18 | 1.133 | 0.244 | 5.264 | 0.875 |
|  |  | Weighted mode | 18 | 1.110 | 0.240 | 5.140 | 0.895 |
| VPA | GE | MR Egger | 10 | 0.556 | 7.2E-04 | 426.115 | 0.867 |
|  |  | Weighted median | 10 | 2.241 | 0.705 | 7.125 | 0.171 |
|  |  | IVW | 10 | 1.977 | 0.853 | 4.585 | 0.112 |
|  |  | Simple mode | 10 | 2.804 | 0.468 | 16.779 | 0.288 |
|  |  | Weighted mode | 10 | 2.554 | 0.405 | 16.099 | 0.344 |
| OAA | GE | MR Egger | 8 | 0.960 | 0.555 | 1.660 | 0.889 |
|  |  | Weighted median | 8 | 1.043 | 0.888 | 1.225 | 0.611 |
|  |  | IVW | 8 | 1.050 | 0.930 | 1.186 | 0.429 |
|  |  | Simple mode | 8 | 1.021 | 0.803 | 1.298 | 0.868 |
|  |  | Weighted mode | 8 | 1.033 | 0.835 | 1.278 | 0.776 |

SNPs, single-nucleotide polymorphisms; OR, odds ratio; LCI, lower confidence interval; UCI, upper confidence interval; IVW, inverse-variance weighted; MPA, moderate physical activities; VPA, vigorous physical activities; OAA, overall acceleration average; EP, epilepsy; FE, focal epilepsy; GE, generalized epilepsy.
