# Supplementary material for: Genetic causal association between physical activities and epilepsy: A Mendelian randomization study
Source: Brain Behav. 2024 Mar 7;14(3):e3463. doi: 10.1002/brb3.3463 (PMC10918602; doi:10.1002/brb3.3463)
Supplement: Supplementary file 3 — Supplementary Table S3. The causal effects of physical activities on the risk of focal epilepsy‐strict definition and generalized epilepsy‐strict definition (without overlap). [file BRB3-14-e3463-s003.docx]

**Supplementary Table S3**. The causal effects of physical activities on the risk of focal epilepsy-strict definition and generalized epilepsy-strict definition (without overlap).

| **Exposures** | **Outcomes** | **Methods** | **SNPs** | **OR** | **95%LCI** | **95%UCI** | ***P-*value** |
| --- | --- | --- | --- | --- | --- | --- | --- |
| MPA | FE-ST | MR Egger | 18 | 21.422 | 0.064 | 7.2E+03 | 0.317 |
|  |  | Weighted median | 18 | 0.817 | 0.239 | 2.796 | 0.748 |
|  |  | IVW | 18 | 0.906 | 0.354 | 2.321 | 0.838 |
|  |  | Simple mode | 18 | 1.021 | 0.111 | 9.420 | 0.986 |
|  |  | Weighted mode | 18 | 1.054 | 0.131 | 8.487 | 0.961 |
| VPA | FE-ST | MR Egger | 10 | 105.746 | 2.898E-05 | 3.9E+08 | 0.562 |
|  |  | Weighted median | 10 | 2.515 | 0.345 | 18.336 | 0.363 |
|  |  | IVW | 10 | 1.272 | 0.201 | 8.039 | 0.798 |
|  |  | Simple mode | 10 | 3.813 | 0.133 | 109.550 | 0.455 |
|  |  | Weighted mode | 10 | 3.124 | 0.142 | 68.756 | 0.489 |
| OAA | FE-ST | MR Egger | 8 | 0.820 | 0.324 | 2.078 | 0.691 |
|  |  | Weighted median | 8 | 0.749 | 0.573 | 0.979 | **0.035** |
|  |  | IVW | 8 | 0.732 | 0.596 | 0.900 | **0.003** |
|  |  | Simple mode | 8 | 0.761 | 0.511 | 1.132 | 0.220 |
|  |  | Weighted mode | 8 | 0.781 | 0.528 | 1.155 | 0.255 |
| MPA | GE-ST | MR Egger | 18 | 0.416 | 0.004 | 45.265 | 0.719 |
|  |  | Weighted median | 18 | 1.220 | 0.485 | 3.073 | 0.673 |
|  |  | IVW | 18 | 1.170 | 0.562 | 2.435 | 0.675 |
|  |  | Simple mode | 18 | 1.265 | 0.234 | 6.843 | 0.789 |
|  |  | Weighted mode | 18 | 1.437 | 0.302 | 6.830 | 0.654 |
| VPA | GE-ST | MR Egger | 10 | 1.020 | 6.9E-04 | 1.5E+03 | 0.996 |
|  |  | Weighted median | 10 | 1.968 | 0.554 | 6.994 | 0.295 |
|  |  | IVW | 10 | 1.769 | 0.701 | 4.463 | 0.227 |
|  |  | Simple mode | 10 | 3.026 | 0.349 | 26.222 | 0.341 |
|  |  | Weighted mode | 10 | 2.478 | 0.298 | 20.571 | 0.423 |
| OAA | GE-ST | MR Egger | 8 | 1.091 | 0.597 | 1.994 | 0.786 |
|  |  | Weighted median | 8 | 1.061 | 0.900 | 1.251 | 0.479 |
|  |  | IVW | 8 | 1.063 | 0.930 | 1.215 | 0.367 |
|  |  | Simple mode | 8 | 1.057 | 0.831 | 1.344 | 0.666 |
|  |  | Weighted mode | 8 | 1.061 | 0.848 | 1.328 | 0.621 |

SNPs, single-nucleotide polymorphisms; OR, odds ratio; LCI, lower confidence interval; UCI, upper confidence interval; IVW, inverse variance weighted; MPA, moderate physical activities; VPA, vigorous physical activities; OAA, overall acceleration average; FE-ST, focal epilepsy-strict definition; GE-ST, generalized epilepsy-strict definition.
